# The struggle for inter-professional teamwork and collaboration in maternity care: Austrian health professionals’ perspectives on the implementation of the Baby-Friendly Hospital Initiative

**DOI:** 10.1186/s12913-016-1336-3

**Published:** 2016-03-14

**Authors:** Christina C. Wieczorek, Benjamin Marent, Thomas E. Dorner, Wolfgang Dür

**Affiliations:** Ludwig Boltzmann Institute Health Promotion Research, Ludwig Boltzmann Gesellschaft, Untere Donaustraße 47, 1020 Vienna, Austria; School of Applied Social Science, University of Brighton, Mayfield House, Falmer, BN1 9PH UK; Institute of Social Medicine, Centre for Public Health, Medical University of Vienna, Kinderspitalgasse 15, 1090 Vienna, Austria; Faculty of Social Sciences, University of Vienna, Rooseveltplatz 2, 1090 Vienna, Austria

**Keywords:** Baby-Friendly Hospital Initiative, Breastfeeding, Collaboration, Teamwork, Health services research, Healthcare professions, Division of labor

## Abstract

**Background:**

The health benefits of breastfeeding for mothers and babies are well documented in the scientific literature. Research suggests that support of breastfeeding during pre- and postnatal maternity care is an important determinant of breastfeeding initiation and duration. To support and promote breastfeeding on maternity units, the Baby-Friendly Hospital Initiative (BFHI) was launched in 1991. In Austria, however, less than one fifth of hospitals with a maternity unit are currently BFHI-certified. Implementation of BFHI and adjunct changes in work practices seem to represent a major challenge to maternity units. This article builds upon previous research that has identified a number of facilitators of and barriers to BFHI implementation in Austria. A major barrier has been the lack of intra- and inter-professional collaboration. Therefore, this article investigates the ways in which different healthcare professionals struggle to work together to successfully integrate the BFHI into practice.

**Methods:**

In this study, a qualitative research approach was used. Thirty-six semi-structured interviews with 11 midwives, 11 nurses, 13 physicians, and one quality manager, working across three maternity units, were interviewed on-site. Data analysis followed thematic analysis.

**Results:**

Midwives, nurses, and physicians had diverse approaches to childbirth and breastfeeding (medicalization vs. naturalness) and worked along different jurisdictions that became manifest in strict spatial divisions of maternity units. In their engagement within the BFHI, midwives, nurses, and physicians pursued different strategies (safeguarding vs. circumvention strategies). These differences hindered inter-professional teamwork and collaboration and, therefore, the integration of BFHI into practice.

**Conclusions:**

Differing approaches to childbirth and breastfeeding, deep seated professional jurisdictions, as well as spatial constraints, challenge inter-professional teamwork and collaboration on maternity units. Inter-professional teamwork and collaboration are widely espoused goals of contemporary healthcare improvement strategies. Yet, critical debate on how these goals can be integrated into practice is needed. To enable collaboration and facilitate the implementation of programs such as BFHI, the different perspectives of health professionals should be brought together and the potential for integrating different forms of knowledge and practices should be considered.

**Electronic supplementary material:**

The online version of this article (doi:10.1186/s12913-016-1336-3) contains supplementary material, which is available to authorized users.

## Background

**Fig. 1:**
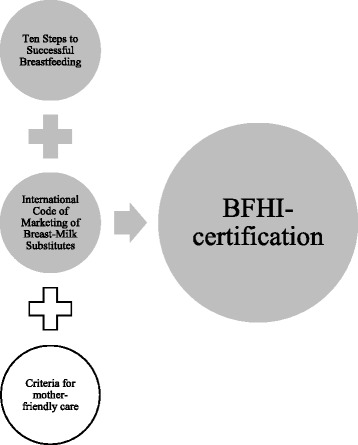
Requirements for hospitals/maternity units in order to gain BFHI-certification

Breastfeeding represents not only the most economical choice for infant feeding, but is also associated with diverse maternal and child health benefits [1, 2]. Moreover, healthcare costs and disease burdens can be effectively reduced by breastfeeding [3, 4]. In recognition of this, the protection, promotion, and support of breastfeeding are major concerns of public health. In 1991, the World Health Organization (WHO) and the United Nations Children’s Fund (UNICEF) launched the Baby-Friendly Hospital Initiative (BFHI), which aims to promote and support breastfeeding on maternity units. With respect to the duration of breastfeeding, WHO recommends at least 6 months of exclusive breastfeeding and continued breastfeeding until 2 years or beyond. To become a “Baby-Friendly Hospital”, hospitals/maternity units have to adhere to a number of requirements as detailed in Fig. 1. While the adherence to the Ten Steps to Successful Breastfeeding (hereafter the ‘Ten Steps’) (also see Table 1 for further clarification of the content) and the International Code of Marketing of Breast-Milk Substitutes (hereafter the ‘Code’) are compulsory for all hospitals around the globe to gain BFHI-certification, the adherence to the Criteria for mother-friendly care depends on decisions of the specific country [5, 6]. Currently, about 20,000 hospitals in more than 150 countries have ever been designated Baby-Friendly [7]. Several studies show that the implementation of the BFHI significantly increases the initiation of breastfeeding and, less strongly, the duration of any and exclusive breastfeeding [8–11]. The latest available data for Austria shows that only a minority of infants are breastfed according to the recommendations of WHO and UNICEF. While more than 90 % of mothers initiate breastfeeding, less than 10 % of babies are still breastfed after 6 months [12]. Data also shows that although the first national hospital earned BFHI-certification in 1996, currently less than one fifth of Austrian hospitals with a maternity unit officially follow BFHI-practices [13]. Compared to other countries, in particular in Northern Europe, these BFHI-certification rates are quite low [14].

**Table 1:** Ten Steps to Successful Breastfeeding [5]

Every facility providing maternity services and care for newborn infants should:	
1.	Have a written breastfeeding policy that is routinely communicated to all healthcare staff.
2.	Train all healthcare staff in skills necessary to implement this policy.
3.	Inform all pregnant women about the benefits and management of breastfeeding.
4.	Help mothers initiate breastfeeding within one half-hour of birth.^a^
5.	Show mothers how to breastfeed and maintain lactation, even if they should be separated from their infants.
6.	Give newborn infants no food or drink other than breast-milk, unless medically indicated.
7.	Practice rooming in - that is, allow mothers and infants to remain together 24 hrs a day.
8.	Encourage breastfeeding on demand.
9.	Give no artificial teats or pacifiers (also called dummies or soothers) to breastfeeding infants.
10.	Foster the establishment of breastfeeding support groups and refer mothers to them on discharge from the hospital or clinic.

^a^Since 2009, this step is interpreted as: “Place babies in skin-to-skin contact with their mothers immediately following birth for at least an hour. Encourage mothers to recognize when their babies are ready to breastfeed and offer help if needed.” [6]

This suggests that the actual implementation of the BFHI presents a major challenge because comprehensive changes are required, especially changes of established practices on maternity units. Therefore, studies that identify facilitators of and barriers to implementing the BFHI are needed. Previous qualitative studies explored health professionals’ perceptions of implementing the BFHI [15–18]; few investigated barriers to and facilitators of BFHI implementation [19–22]. In Austria, a needs assessment among experts showed that the experts generally recognize the relevance of the BFHI and advocate an expansion of BFHI-certification rates, at least from a public health perspective [23]. To promote BFHI-certification in Austria, the Austrian Federation financially supported multiple roll-out actions over the last 3 years. These actions followed the new organizational set-up of the BFHI. While in the beginning, UNICEF Austria supported the BFHI and a few individuals carried out BFHI-certification, since 2010, a specialized BFHI division of the Austrian Network of Health Promoting Hospitals and Health Services coordinates and carries out BFHI-certification. Since then, Austrian hospitals do not only have to follow the Ten Steps and the Code, but they also have to adhere to the Criteria for mother-friendly care to become a Baby-Friendly Hospital, as suggested by WHO and UNICEF. For an overview of the specific mother-friendly care criteria in Austria see Table 2.

**Table 2:** Criteria for mother-friendly care in Austria [13]

A natural birth experience is a significant prerequisite for successful breastfeeding. Therefore, mother-friendly care is a compulsory part of BFHI-certification. The criteria require, unless medically indicated that:	
a)	Mothers can bring a companion of their choice to provide continuous physical and/or emotional support during labor and birth, as desired.
b)	Mothers can drink and eat light foods during labor, as desired.
c)	Mothers can walk and move about during labor, as desired.
d)	Mothers can choose a position while giving birth.
e)	Mothers should be offered the use of non-drug methods of pain relief.
f)	Invasive procedures such as rupture of the membranes, episiotomies, acceleration or induction of labor, instrumental deliveries, or caesarean sections should be used only for medical indications.
g)	Standards, guidelines and training curricula of the maternity unit support mother-friendly care.

In a climate of the increased attention given to BFHI in Austria over the last few years, we recently conducted a qualitative study among health professionals on their views on and attitudes to BFHI implementation [24]. In our previous analysis, we identified several activities that our maternity units took in order to facilitate the implementation of BFHI. Examples of such activities comprise (i) consensus building with the managers of all relevant professional groups as well as hospitals’ top-management, (ii) building an inter-professional BFHI project group as emphasized by the Austrian BFHI division, (iii) training activities etc. [24]. Besides this, our preceding analysis showed that a number of barriers had to be overcome for more widespread implementation. Among mothers and their relatives, analysis indicated that language and literacy barriers as well as expectations about birth and breastfeeding hindered BFHI-implementation. Among staff, barriers included, for example, lack of time and staff resources, old professional patterns, personal experiences, as well as a lack of intra- and inter-professional collaboration [24].

In line with our findings outlined above, previous studies emphasized the relevance of health professionals’ personal characteristics impacting on BFHI implementation. Semenic et al. [20] in an integrative review of BFHI implementation and, more recently, Schmied et al. [25] in a meta-ethnographic synthesis, note that individual beliefs and attitudes of health professionals considerably influence implementation. Similarly, Greenhalgh et al. [26] raise concerns about the lack of attention given to understanding personal characteristics of staff as determinants of change in health service delivery and organizations.

While a number of studies investigated professionals’ perceptions of BFHI implementation and its impact on practices, a more in-depth understanding of diverging views of health professionals can be gained by comparing their experiences. In our previous research [24], we noted that multiple barriers have to be overcome to put the BFHI into practice on the maternity unit. One major barrier identified was the lack of inter- and intra-professional collaboration. Data indicated that differences within and between professionals working on maternity units impeded BFHI implementation. In this paper, we extend our previous analysis by acquiring a deeper understanding of factors that can explain health professionals’ struggle to promote and support breastfeeding on maternity units in a collaborative manner. To do so, we investigated the following questions:

1. Which approaches do health professionals have to childbirth and breastfeeding?
2. How does collaboration work in the face of professional and spatial boundaries?
3. Which strategies do health professionals pursue in their engagement within the BFHI?

## Methods

### Study design

For this study, a qualitative approach based on individual, semi-structured interviews and “thematic analysis” following Ritchie and Lewis [27] was chosen as a method for interpreting the data. This approach is well-suited for exploring individuals’ views and experiences of concrete phenomena as well as abstract concepts [27]. In our case, we applied this approach to investigating health professionals’ perceptions of the implementation of BFHI.

### Setting

In Austria, there is a mandatory insurance system and hospital care is funded publicly. Maternity and child care is provided at 79 maternity units, distributed around the country. Municipalities, provinces, as well as social insurance companies are the main owners of hospitals. However, there are also some privately owned hospitals, such as those run by religious orders, as well as a few private, for-profit hospitals where women can deliver at their own expense. Over the last decade, birth rates have been quite constant. Data from 2012 indicates that in Austria, 78,952 infants were born [28].

Our study took place in three hospitals/maternity units in one urban area of Austria. Each maternity unit had public access for women with the obligatory health insurance. Two of the hospitals had a public owner and one a private owner (a religious order). Annually, the three units together had about 6,000 births and only 4.3 % (254) of mothers had ambulatory births in 2012. With respect to BFHI-certification status, two hospitals had already been BFHI-certified over the previous 2 years and one was working towards its first BFHI-certification at the time of our study. Following Austrian requirements for BFHI-certification, as formulated by the BFHI division of the Austrian Network of Health Promoting Hospitals and Health Services, BFHI-certification needs to be refreshed every 4 years.

### Study participants and recruitment

Purposive sampling [27] was used to select study participants. This sampling strategy allowed us to generate rich information for our study on health professionals’ perceptions of BFHI implementation as well as gather a range of opinions. Based on consultations with experts in the field, we included 35 health professionals as well as one quality manager who appeared to play a major role in the installation of the BFHI in one of the hospitals. In particular, in each hospital, the managers of midwives, nurses, and physicians were contacted and asked to participate as well as to inform and motivate at least two colleagues, preferably with different years of work experience, to participate in the study. We intended to recruit staff from different professional backgrounds (midwives, nurses, pediatricians, gynecologists, and anesthesiologists) working in management positions as well as non-executive positions. The lead author (CCW) contacted each designated participant individually on the phone to provide further information about the study, to get agreement to participate and, for those who consented, to schedule a date for the interview. To minimize recruitment pressure among employees in non-executive positions, only those who were available and willing to participate in the study were included. Accordingly, none of the contacted professionals denied participation. Overall, the group of interviewees included 11 midwives, 11 nurses, 13 physicians, and one quality manager. Concerning the group of physicians, eight gynecologists, three pediatricians, and two anesthesiologists participated in the study. The majority of the participants were female. Male participants were the quality manager and two physicians in management positions. In all hospitals, the physician manager was also the head of the whole maternity unit. For a detailed overview of the participants’ profiles see Table 3.

**Table 3:** Overview – participants’ profile

Participant and occupation	Gender		Position		Years of work experience		
	Female	Male	Management	Non-executive	<5 years	5–15 years	>15 years
Hospital A^a^ (*N* = 14)							
Physicians (*N* = 5)	5		1	4	1	2	2
Midwives (*N* = 5)	5		1	4		2	3
Nurses (*N* = 4)	4		1	3		1	3
Hospital B^a^ (*N* = 11)							
Physicians (*N* = 4)	3	1	1	3		2	2
Midwives (*N* = 3)	3		1	2	1		2
Nurses (*N* = 4)	4		2	2		1	3
Hospital C (*N* = 11)							
Physicians (*N* = 4)	3	1	1	3		2	2
Midwives (*N* = 3)	3		1	2	1		2
Nurses (*N* = 3)	3		1	2		3	
Quality manager (*N* = 1)		1	1			1	

^a^Already BFHI-certified

### Data collection

Between August and December 2013, 36 semi-structured interviews were conducted on-site. The average length of each interview was 1 h and for each interview, two researchers (CCW and another male senior researcher) were present. During the interviews, we used an interview guide that provided narrative stimuli as well as the topics that were to be covered (the original interview guide can be found in the Additional file 1). The main themes of the interview guide followed the three main stages of implementation, which we had defined for the purpose of our study, and which are based on logical models of implementation in organizations [29, 30] as well as the systematic review by Greenhalgh et al. [26]. These included *(1) Selection of the BFHI, (2) Installation of the BFHI, (3) Operation of the BFHI* (for further details compare [24]). Questions asked included:

- What is your role in relation to the implementation of the BFHI?
- What are your general views on and opinions about the BFHI?
- What are the challenges that your hospital experienced in becoming Baby-Friendly?
- How were barriers overcome?

The interview guide was used by the interviewers as a memory aid and was not meant to strictly structure the interview according to a certain order. The first four interviews – all conducted in hospital A – were used as pilot interviews in order to assess the scope of the interview guide and to ensure that interviewees were given sufficient opportunities to provide coherent accounts of their central issues on BFHI implementation [27]. Afterwards, experiences with the interview guide were discussed within the research group (CCW, BM, TED, WD and another senior researcher) and it was agreed that interview guide helped to ensure that key issues were tackled. At the same time, it was also concluded that it facilitated flexible investigation.

All interviews, except one for which the interviewee refused recording, were audio-taped and transcribed verbatim. For the one exception, we wrote field notes. Confidentiality was ensured by using pseudonyms for interviewees in all paperwork and types.

Once the last interview in hospital C was finished and because data analysis was already started, the interviews as well as the preliminary findings of the data analysis were discussed in the research group (CCW, BM, TED, WD and another senior researcher). It was agreed that we could stop data collection because we had reached a stage where we were predicting health professionals’ responses and where no new information was provided by the interviewees [27].

### Data analysis

Interviews were analyzed using thematic analysis [27]. To familiarize ourselves with the data, we read through the transcripts several times. Identification and labeling of codes followed the main themes of our interview guide, as well as the themes which appeared in the data. Within the thematic clusters generated, we identified health professionals’ views and attitudes on childbirth and breastfeeding, as well as their views on collaboration in practice, and, finally, strategies to enable BFHI implementation as major themes and further condensed these. Afterwards, we undertook interpretations of each theme and summarized the main findings. To compare similarities and differences between the professional groups, we sub-divided our summaries for midwives, nurses, and physicians respectively. These allowed for investigation of the unique elements of each professional group. The first and second author (CCW, BM) carried out all of the analysis and they also discussed and negotiated the interpretations on a weekly basis. To further enhance the rigor of the data analysis, the first and second author debated preliminary outcomes and interpretations with the whole group of researchers (with TED, WD and another senior researcher) in a series of meetings. These meetings took place over a period of six months and aimed to reach consensus on the ultimate interpretation of the data. Furthermore, we summarized major findings point-by-point and e-mailed them to the participants in order to discuss and confirm preliminary conclusions drawn by the research group. Atlas.ti was used to support the management and analysis of the data.

### Ethical issues

Before commencement of the study, the Ethics Committee of the City of Vienna gave approval (EK 13-188-VK_NZ). In advance of the interviews, each participant was informed that participation was voluntary and confidentiality would be ensured at all stages of the research process. In this regard, we obtained written informed consent from each participant. Participants had the option of turning off the recorder or stopping the interview at any point in time.

## Results

The depiction of findings follows salient themes and sub-themes which the health professionals reported in relation to their views on and attitudes to childbirth and breastfeeding, their views on collaboration in practice, and strategies employed to enable BFHI implementation. To illustrate how the interpretations are grounded in the data, we use direct quotes in a conversational format for each theme and sub-theme and include a code number for each interviewee after the quote (Midwives: M1 to M11, Nurses: N1 to N11, Physicians: P1 to P13, Quality manager: Q1). Table 4 provides further quotes of the interviewees to allow more insight in our data.

**Table 4:** Additional quotes from interviewees

Theme	Sub-theme	Professional group	Verbal quote
Theme 1: Health professionals’ approaches to childbirth and breastfeeding	Medicalization of childbirth and breastfeeding	N7	… and of course, sometimes you intervene, you can’t help it.
		N8	… one is used to give a bottle when mothers say they think to have insufficient breastmilk, because then breastfeeding problems are settled. Convincing mothers and supporting them that they don’t stop, but rather continue, that’s really difficult.
		M11	There are nurses, child nurses [on the ward]. There are lactation counselors, those who are IBCLC-certified, but often it’s these strict rules rather than caring on an individual basis…
		P12	Of course, I’m convinced about the benefits of breastfeeding and women should possibly be supported to be able to breastfeed. Even after discharge, they should still wish to breastfeed rather than stopping it because they’re too stressed. It’s good […] for their [babies’] physical condition. For the immune response and the like and for mothers…
		M8	Besides, I’ve the impression that nipple shields are used quite fast and quite often… I don’t know the specific reasons, whether it’s then easier for nurses to support breastfeeding.
	Naturalness of childbirth and breastfeeding	P1	Midwives also provide reasons for it [BFHI] and [they outline] that it’s very important for the bonding between mother and baby, that this is really substantial. Concerning antibodies, we know from medicine that it’s relevant… [Midwives emphasize] again and again, that it’s crucial for the close relationship between mother and baby.
		M8	If the mother has delivered under my supervision and if I’ve seen that breastfeeding works without nipple shields but this [giving nipple shields] will be the first intervention after 3 h, I’ll go to the nurse and ask her directly why this is necessary?
		M3	This whole process starts with increasing salivation among babies. Fascinating, really and due to this oozy cheeks they can slip to it, I mean, you have to consider this, how genial nature is.
		M10	Give [mothers] security that nature has prepared them. Of course there are sometimes exceptions, that not every mother can [breastfeed].
Theme 2: Collaboration in the face of professional and structural boundaries	Professional jurisdictions	N5	After c-section, you need the anesthesiologist, the gynecologist, the pediatrician, the midwife, sometimes an additional nurse … The problem about this is again, these habitual jurisdictions…
		N7	Because when there are lactation problems, then you’ve to ask for a gynecologist, because this problem is a problem that relates to mothers, thus, mother issues, they relate to gynecologists.
		P7	Because it [delivery] changes the focus, I become less and less interesting as gynecologist, before, I’m the most interesting person for the mother.
		P10	Following our system, it’s midwives who are responsible for the labor room and who take care of mothers up to a few hours after delivery. Then mothers will be handed over to nurses. From then on, nurses are responsible for taking care and counselling.
		M10	… there is some little exchange [between midwives and nurses] when moving mothers from the labor room to the ward. Otherwise, there is hardly any exchange, well, we’re really separated divisions.
		P7	… because we don’t have any executive power. Anesthesiologists have their work area and are responsible for this area. In the beginning they said, no one is allowed to enter the operating theatre because they are responsible. In the end, you run against a wall and you can’t overcome it because it’s right, it’s his [the anesthesiologist’s] area.
		P2	As we’re [gynecologists] only responsible for ward rounds on the maternity ward and as we aren’t present on the ward the whole day, it [breastfeeding counseling] belongs to the responsibility of the respective nurse…
		P10	As long as they [mothers] have no baby, I’m not involved as a pediatrician. I don’t know, we’ve discussed repeatedly how much of an issue breastfeeding is once women reach the end of their pregnancy.
	Spatial division of maternity units	N9	They’re really isolated in their labor room although the labor room and the ward are next to each other. To get to the labor room, you even have to pass the ward, but still, we don’t know every name of every midwife… there isn’t a strong connection.
		P13	Because during daily work there outside [on the ward], I think…
		M10	But it would make sense to handover all issues which we’ve [midwives] already explained to her [the mother], which breastfeeding position we’ve shown, thus a little bit more elaborated. Also outside on the ward, … there should be more explicit handovers
Theme 3: Strategies to harmonize professional approaches with BFHI implementation	Safeguarding and defense strategies	N2	… you have a standard which specifies who does what, where does he/she conduct it, when is it conducted.
		N6	If you don’t have any standards or any points of reference or how shall I say, guidelines, it’ll slip somehow and after a while you’ll return to old practices.
		M10	Well it’s just, probably to shape it consistently, there are standards, and then the individual interpretation is often neglected [by nurses]. Well, I hardly appreciate that.
		M11	For me, Baby-Friendly means to act in an individual manner, but… nurses want to hear: if this than that, and that. But this isn’t applicable to breastfeeding and maternity and child care.
	Circumvention strategies	M3	However, even in my case, there are aspects [of BFHI] which I refuse. For example, the standard or guideline that every woman has to get skin-to-skin contact directly after c-section, I cannot sign this.
		N9	… the anesthesiologists, they think, it [BFHI] isn’t relevant to them… they think it [BFHI] won’t bother them.
		P1	I can only remember that during one night shift it was said at 3.00 am, that we’ve to do skin-to skin contact. I’ve to say, I’ve denied it. Everything was so exciting and the father ran around
		P1	Well we, the anesthesiologists hardly have to do anything with it [BFHI)…
		N9	Well, it’s, midwives are really self-confident, it’s a really self-confident professional group.
		P13	The team of gynecologists is quite fragmented. Really fragmented, there is a break between advocates and refusers, i.e. physicians who don’t feel responsible for breastfeeding.

### Theme 1: Health professionals’ approaches to childbirth and breastfeeding

Data analysis revealed that one challenge to achieving the necessary inter-professional teamwork to integrate BFHI into practice is the differing approach of midwives, nurses, and physicians to childbirth and breastfeeding. Two sub-themes emerged: ‘medicalization of childbirth and breastfeeding’ and ‘naturalness of childbirth and breastfeeding’.

#### Medicalization of childbirth and breastfeeding

Following the accounts of interviewees, nurses and physicians, in particular, displayed a largely bio-medical approach to childbirth and breastfeeding. They defined childbirth and breastfeeding in medical terms and described these processes as requiring medical control. Generally, while nurses and physicians saw the benefits of breastfeeding, they advocated it in terms of its nutritional properties, as well as its health benefits, including reduction of infectious diseases or improved child growth. In this sense, a pediatrician stressed: “*I’m a breastfeeding supporter. I believe that breast-milk is the most suitable diet for newborns. For 100 %. I just see that it* [breastfeeding] *has documented health benefits for all babies.*” (P10) As illustrated by this comment, nurses and physicians typically considered breast-milk to be the superior infant food. Next to the health benefits of breastfeeding for the child, nurses, and physicians were also aware of the evidence on the health benefits of breastfeeding for the mother. Here, they referred to the faster return to pre-pregnancy weight or to reduced breast- and ovarian cancer rates. Accordingly, nurses and physicians described the BFHI as a valuable and desirable intervention that positively affects the health of babies and mothers (also see P12 in Table 4).

Data further showed that nurses and physicians adopted rather paternalistic and interventionist practices to support and promote childbirth and breastfeeding. Both, nurses and physicians mentioned that they are used to surveillance and control of breastfeeding rather than enabling mothers to accomplish and monitor breastfeeding by themselves. Nurses explained that they were used to actively providing care to mother and child. Both physicians and midwives perceived nurses as “*overprotective*” and pointed out that nurses had difficulties holding back and having a supportive and mentoring function for mother and child as emphasized in the BFHI (see Ten Steps summarized in Table 1). This interventionist approach of nurses becomes evident in the following statement: “*It’s just in my case, as a nurse, you routinely tend to intervene instead of observing how the child and the mother are interacting and what they can achieve by themselves.*” (N7) Consistent with this comment, nurses’ interventionist practices with breastfeeding were a major issue of concern among midwives. Midwife interviewees often criticized nurses for their interventionist practices and for being too likely to use various technologies to support breastfeeding such as breast pumps and nipple shields (see M8 in Table 4).

Interventionist practices were also common among physicians, in particular pediatricians. Nurses and midwives explained that pediatricians had difficulty with the idea of breastfeeding on demand (step 8). In this respect, interviewees said that pediatricians tended to measure the success of breastfeeding (largely) on the basis of pediatric reference points for infant weight gain. Another frequently discussed topic with regard to pediatricians’ interventionist practices was the use of pre- and post-feeding weights. This involves the measurement of the baby’s weight before breastfeeding and after breastfeeding to monitor how much breast-milk has been transferred to him/her during the feed. Pre- and post-feeding weights were recorded, in particular, because pediatricians doubted that mothers could produce sufficient quantities of breast-milk. Moreover, our study revealed that pediatricians were concerned more about the quantity rather than the quality of breast-milk, such as its possibly being contaminated. By contrast, several midwives and a few nurses who strongly advocated breastfeeding pointed out that newborns’ stomachs are quite small, and, in this context, even a drop of breast-milk may be almost sufficient. However, findings in one of the already BFHI-certified hospitals revealed that struggles among professionals about pre- and post-feeding weights were reduced after the introduction of BFHI. One midwife explained that following the introduction of BFHI, the usage of pre- and post-feeding weights almost completely stopped: “…*simply these stupid pre-post feeding weights: according to certain criteria, you have to provide formula feeding… what really happened* [after introducing BFHI]*, these pre-post feeding weights were eliminated, more or less.*” (N3) As this comment shows, BFHI implementation is not only hindered by differing approaches towards childbirth and breastfeeding among health professionals, but BFHI can also initiate some changes and ease collaboration.

#### Naturalness of childbirth and breastfeeding

By comparison to nurses’ and physicians’ medicalized approach to childbirth and breastfeeding, midwives largely refer to the naturalness of these processes. Therefore, midwives are very supportive of BFHI requirements, such as breastfeeding on demand (step 8) or the avoidance of formula feeding unless medically indicated (step 6). During the interviews, midwives emphasized their professional expertise and their positive attitude to childbirth and breastfeeding. As such, one midwife stated: “*Breastfeeding is an integral part of our being* [as midwives].” (M3)

In describing their work, midwives referred to person-centered practices that place the mother and the baby in the forefront. Accordingly, midwives considered BFHI to be a valuable and desirable intervention because it strengthens the relationship between mother and baby. Data analysis also revealed that midwives largely employed a discourse that pays particular attention to working collaboratively with the mother and emphasizes the normal physiology of childbirth and breastfeeding: “…*we, midwives, consider pregnancy, childbirth as well as postpartum period as something physiologically healthy. Yet, although most of the nurses know that the mother and her baby are healthy, they often assume that they have to interfere and have to provide care, which is often not necessary.*” (M11) This comment illustrates the midwives’ perception that the essential first step in caring for the mother and child is to observe their interactions. Nurses and physicians also acknowledged that midwives try to avoid intervening in the processes of childbirth and breastfeeding (see P1 Table 4). The relevance of trusting in the naturalness of childbirth and breastfeeding also becomes evident in the following comment: “…*one has to trust in the babies, trust them that they know what to do and the same holds for the mothers*…” (M9) Moreover, midwives explained that considering breastfeeding as a natural physiologic process means that they primarily have to give emotional support to mothers and encourage them to trust in their lactating bodies. The significance of empowering mothers to trust in their bodies as well as building mothers’ confidence in relation to breastfeeding was described as one major task of midwives’ work. Midwives emphasized that mothers should be supported in building trust that nature has prepared them to feed their babies effectively rather than having midwives take over the responsibility for the babies from their mothers. In line with these perceptions, midwives felt that supporting breastfeeding requires protecting mothers from unnecessary medical treatment, intervention, and interference, such as the provision of breast pumps or nipple shields (see M8 in Table 4).

Another important aspect, typically highlighted by midwives, was individualism. Midwives explained that each mother and each child is unique and, therefore, supporting childbirth and breastfeeding requires individualized practices. They emphasized the importance of following mothers’ and babies’ cues and habitual practices, as well as considering the reciprocal relationship between mother and child: “*If mothers have breast pain, nipple pain because of latching on, latching on, latching on, it’s necessary to be mother-friendly and you have to do that. You have to balance latching on against mothers’ needs.*” (M11) This quote illustrates midwives’ perceived relevance of implementing the BFHI on an individual basis rather than a strict by-the book integration of BFHI. In particular, the midwife here refers to step 8 “Encourage breastfeeding on demand” which should be balanced against the needs of the mother.

As we will see in the following, the co-existence of different approaches to childbirth and breastfeeding has considerable implications for working together and collaborating on the BFHI.

### Theme 2: Collaboration in the face of professional and structural boundaries

The analysis of health professionals’ views on struggles to integrate BFHI into work routines revealed two interrelated sub-themes: ‘professional jurisdictions’ and ‘spatial division of maternity units’.

#### Professional jurisdictions

Characteristically, midwives, nurses, and physicians agreed that maternity and child care and, in this sense, also the integration of BFHI into work practices, require extensive collaboration and communication among their professional groups. Participants emphasized that to integrate BFHI they have “*to pull together*”. However, data showed that this shared position did not equate to agreement on *how* to integrate BFHI into practice. To illustrate this, interviewees continuously referred to enabling skin-to-skin contact after cesarean section (c-section) (also see N5 Table 4). They commented that this is a significant situation where all professional groups come together and must collaborate. While explaining the difficulties, health professionals emphasized their fixed role assignments and established definitions of their scope of responsibilities. One nurse said: “…*everyone has his jurisdiction.*” (N4) As such, nurses and midwives mentioned that it was particularly difficult to encourage colleagues from another professional group to enable skin-to-skin contact after c-section: “…*it’ll only work if professional groups find a good consensus among each other. In this way a professional group has to respect that another group* [in this case, anesthesiologists] *says, e.g., that they want to see mothers’ skin.*” (M3) Here, the midwife stresses that, even in the context of BFHI, one has to accept that, for anesthesiologists, monitoring mothers’ skin outweighs enabling skin-to-skin contact. Yet, it also became clear that such competing professional jurisdictions made the integration of BFHI into work routines extraordinarily challenging (e.g. P7 Table 4).

Within the data, clear-cut role assignments were repeatedly emphasized by all professional groups. Midwives explained that they are primarily responsible for the mother before and during birth as well as up to 2 h after birth. In relation to the BFHI, data analysis showed that midwives had mostly taken over pre-natal breastfeeding counseling duties as well as enabling the first breastfeeding after birth. Thus, data indicated that midwives were able to expand their fixed role assignments as the example of taking the responsibility for the child after c-section shows. However, midwives were also critical about promoting skin-to-skin contact after c-section. Midwives highlighted in the interviews that they do not want mothers to accept c-section as a normal natural birth as a result of the introduction of skin-to-skin contact. In this respect, one midwife commented: “*I’m afraid that mothers will get the impression that c-section is a ‘good’ childbirth.*” (M1) As this illustrates, many midwives were concerned that enabling skin-to-skin could hide the fact that c-section is an unnatural form of birth, which should only be used in urgent cases.

The data further revealed that postnatal counseling activities are typically undertaken by nurses on the ward (also see P10 Table 4). Here, midwives, nurses, and physicians agreed that established care concepts such as ‘family nursing’ facilitated collaboration and BFHI integration into practice: “*In this hospital, every nurse is responsible for both mother and child. In other hospitals, nurses caring for mother-child dyads aren’t necessarily state of the art. They don’t have this mutual care concept.*” (M2) Midwives and physicians, but also nurses themselves evaluated family nursing positively. They emphasized that collaboration with nurses was eased to some degree because other health professionals had only one contact person for each mother-baby dyad.

Physicians’ role assignments were described as clear-cut territories. The data showed that it was difficult to integrate BFHI into their territories. In particular, gynecologists and anesthesiologists typically referred to medical issues, such as monitoring and surveillance before and during birth. By comparison, BFHI-related activities, such as the promotion of breastfeeding and counseling activities, were primarily seen as tasks for midwives and nurses and represented a very small component of physicians’ activities (P2 and P10 Table 4). As highlighted in Table 4, a gynecologist (P2) explained that they become “uninteresting” once the child is born, anesthesiologists explained that they are relevant during enabling skin-to-skin contact after c-section. On the other hand, midwives and nurses repeatedly described anesthesiologists impeding collaboration and hampering BFHI implementation: “*Concerning other professional groups, well, I mean there’s one group, you can say, they’re 100 % adversaries. It’s only a small group…, it’s the anesthesiologists. I really don’t know anyone who supports it* [the BFHI]*. I can’t believe it…*” (M4) Anesthesiologists did not see the advantages of BFHI and rather felt disturbed when it came to enabling skin-to-skin contact after c-section because the administration of medication as well as monitoring mothers’ skin are impeded. Nevertheless, several midwives as well as few nurses who strongly advocated breastfeeding stressed that it was the anesthesiologists’ stubbornness rather than any serious medical disadvantages of having the baby in skin-to-skin contact. By comparison, pediatricians were considered to be the group of physicians that was most likely to get in touch with breastfeeding and breastfeeding problems. In this regard a gynecologist explained: “*As a pediatrician you’re more likely to see the advantages of breastfeeding as compared to any other professional group. Gynecologists, like I am, for example, don’t see it* [breastfeeding benefits]*. They only see whether it* [breastfeeding] *works or not but they won’t see the long-term benefits.*” (P13)

#### Spatial division of maternity units

Beyond professional jurisdictions hindering collaborative practices, data analysis revealed several structural aspects as hindering factors. In this regard, interviewees referred to the spatial division of maternity units into labor room, maternity ward, and nursery. The impact of the spatial division became particularly evident in interviews with midwives and nurses (N9 Table 4). They repeatedly made an inside-outside division between the labor room (inside) and the ward (outside). One nurse pointed out: “*It’s unacceptable that mothers inside in the labor room get different information* [with respect to breastfeeding management] *compared to what they learn from us outside on the ward.*” (N11) This comment indicates that, due to the spatial division, there is a lack of communication and consistent provision of information about breastfeeding. The impact of the spatial division between midwives and nurses becomes evident with the use of phrases such as, “*from us outside*”. By using ‘inside-outside’ metaphors, nurses and midwives underlined the difficulties of collaboration and exchange. Yet, not only nurses and midwives made these inside-outside division, but also some physicians, while talking about post-natal breastfeeding counseling, referred to the nurses “*there outside*”.

Data further showed that the spatial division appeared to hinder effective and timely sharing of knowledge and expertise (M10 Table 4). While explaining that the spatial division of maternity units hardly left space for inter-professional exchange, midwives and nurses especially referred to the lack of inter-professional meetings. As a consequence, BFHI was also discussed in a mono-disciplinary way, as one midwife mentioned: “*We’ve done it* [the exchange of information] *during team meetings. Midwives and nurses did it during their team meetings and physicians during their team-Wednesday* [where information on BFHI was shared].” (M2) As this midwife points out, there was little collaboration and exchange among midwives, nurses, and physicians in managing childbirth and breastfeeding.

To overcome the spatial constraints of maternity units, the implementation of BFHI facilitated new forms of meetings. In all cases, an inter-professional project group and sub-groups were built to work collaboratively towards BFHI-certification. Nevertheless, data in the two hospitals already BFHI-certified showed that, as soon as BFHI-certification was achieved, inter-professional meetings declined and almost disappeared. In this sense, one midwife explained: “*I think it is* [BFHI integration into daily practice] *happening in waves. It really depends on the point within the BFHI-certification phase. If BFHI-certification is close, enormous efforts are made and something is really happening. After BFHI-certification is completed, accuracy decreases and people hardly care about it.*” (M4) This quote shows that inter-professional exchange remains time-limited and has not been able to be sustainably established on maternity units.

### Theme 3: Strategies to harmonize professional approaches with BFHI

Data analysis revealed that health professionals characteristically pursued a range of strategies by their engagement within the BFHI and these diverging strategies seemed to impede collaborative practices and, thus, BFHI implementation. The most salient strategies involved ‘safeguarding and defense strategies’ as well as ‘circumvention strategies’.

#### Safeguarding and defense strategies

According to the data analysis, nurses considered BFHI as an opportunity to safeguard established practices by uniting their professional approaches with BFHI. Nurses indicated that newly developed Baby-Friendly standards helped them to better justify their work vis-à-vis midwives and physicians. As compared to midwives and physicians, data analysis showed that nurses very strictly adhered to the requirements of the BFHI (also compare N2 and N6 Table 4). Nurses themselves often explained that they use the backing provided by written Baby-Friendly standards, such as breastfeeding guidelines, to justify their work. They considered such guidelines useful because they outline (a) the precise duties of each professional, (b) the specific moment in time to conduct particular activities, and (c) the place to fulfill certain duties. Referring to this, one nurse explained: “*The advantage is, I think, that if you implement it* [the BFHI] *you’ll have clear guidelines at hand*…” (N3)

However, midwives were often critical of the way nurses implemented BFHI. Midwives emphasized that Baby-Friendly standards should be used in a flexible manner and should be interpreted and adapted to the specific situation and condition of mother and child rather than in a strict and universal manner (see M10 and M11 Table 4). Midwives were concerned that nurses tend to neglect the naturalness of childbirth and breastfeeding. A physician supported this perception and commented: “*Yet, nurses do their job and they really pursue it* [BFHI] *with a lot of vehemence.*” (P8) While nurses’ vigor in integrating the BFHI into practice often raised concerns, one physician also argued that there are certain reasons for nurses’ behavior: “*I think they* [nurses] *are pressurized a lot. I have the impression that they are the poorest within this system. They are under much pressure, they have a strong hierarchical system and I think this is enacted very strictly.*” (P12) This observation reveals that, from the perspective of this physician, nurses are in a weaker position as compared to other professional groups. Midwives supported this view, as the following statement makes clear: “*Nurses always need regulations and orders whereas we* [midwives] *hardly need regulations and orders… A nurse can’t do anything alone* [without assignment]… *Moreover, we have often had problems with pediatricians because they have tended to interfere in our work.* [As a midwife] *you really feel disturbed in your work.*” (M1)

#### Circumvention strategies

Similar to nurses, midwives promote and support BFHI and its underlying principles. However, by comparison to nurses, midwives were rather critical about the content of BFHI as well as its integration into practice. They were concerned that BFHI follows a too medical and technical approach, and that the mothers’ needs are shortchanged. Therefore, they largely used strategies to advocate mothers’ self-determination and need to rest. Midwives, then, considered BFHI requirements as a reference system that should be continuously adapted to the specific situation and the emotional and physical needs of mothers and babies. One midwife explained: “*If I have the impression that breast-milk will come, I can say we’ll wait although the baby has already lost a lot of weight. But I take care of that, it’s my responsibility. Yet this* [waiting although baby has lost a lot of weight] *isn’t possible outside* [on the ward with the nurses].” (M10) As this comment indicates, midwives had the courage to circumvent formula feeding, although from a medical perspective it would be suitable (see step 6). Midwives, compared to nurses, claimed their own professional knowledge and therefore called for the individual application of the BFHI (also compare M3 Table 4). Midwives themselves, but also nurses and physicians, felt that midwives have “*strong characters*” and do not hesitate to deviate from written standards. However, midwives’ individual interpretation and adaptation of the BFHI, as well as their recurrent circumvention practices, raised concerns among nurses: “*There are so many stories, but I don’t want to go into too much detail… but sometimes they* [midwives] *really believe they can do everything. Yet, alas, I’ve to say, this is often not the case.*” (N4) This observation illustrates the claims for professional autonomy by midwives who do not shy away from conflict with nurses or physicians in order to impose their individual interpretation of BFHI and its requirements.

Data analysis further revealed that physicians also tend to circumvent BFHI. By contrast to nurses and midwives, physicians merely accepted the BFHI because they acknowledged the positive health benefits for mother-baby dyads. Yet, BFHI was only accepted as long as the physicians’ work and their jurisdictions remained untouched. Data showed that, generally, physicians tried to reduce their involvement to a minimum. One gynecologist even explained: “*These* [BFHI] *guidelines make it difficult to consider the individuality. For myself, this is a critical point and because of that I have recently tried to avoid ward rounds…*” (P12) As this comment shows, this gynecologist sidesteps ward rounds because (her perception is that) BFHI requirements prevent the physician from taking care of the mother individually. Moreover, physicians place great weight on their established professional territory and try to defend this territory from inroads made by BFHI. In this way, one midwife in a hospital already BFHI-certified reported: “*In the beginning, some anesthesiologists really resisted bonding after c-section. They were cutting off the tubes for enabling skin-to-skin contact* [a tube where babies could be placed to prevent falling down] *and were saying ‘we don’t need it’.*” (M6) As this indicates, physicians showed a lack of interest and support and rather defended their own territory. Nurses and midwives noted that, ultimately, there was very little they could do to influence physicians’ medical practice.

## Discussion

The triad of midwives, nurses, and physicians provides a unique lens to investigate the complexity of inter-professional teamwork and collaboration on maternity units. In this study, we have shown that midwives, nurses, and physicians have overlapping and diverging approaches to childbirth and breastfeeding and professional jurisdictions and encounter spatial constraints that impede the integration of BFHI into practice. Investigating commonalities and differences as well as analyzing apparent conflicts between professional groups is necessary to better understand how BFHI can be successfully implemented in a collaborative manner.

While previous studies on the implementation of BFHI highlight the relevance of inter-professional teamwork as well as collaborative approaches to enabling BFHI implementation [17, 19, 20, 24, 25], they pay little attention to how professional identities and boundary work advanced by midwives, nurses, and physicians play out in maternity care that follows BFHI requirements. Therefore, this article explored the views and attitudes of midwives, nurses, and physicians in relation to collaborative practices and structures.

The co-existence of different approaches to childbirth and breastfeeding and competing claims for knowledge has considerable implications for the integration of BFHI-related activities into professionals’ work routines. Our findings clearly indicate that nurses and physicians share a bio-medical and interventionist approach to childbirth and breastfeeding and therefore, interpret BFHI requirements differently from midwives. Due to their bio-medical knowledge base and interventionist work practices, both nurses and physicians interpret the BFHI in medical terms and tend to interfere in childbirth and breastfeeding. Yet, midwives disapprove these interventionist practices. By emphasizing different philosophical stances and practices among various professional groups, our study provides an in-depth understanding about factors that can inhibit collaboration and hinder the integration of BFHI into practice. Thereby, we provide further insights that can explain health professionals’ struggle to collaboratively implement the BFHI in Austria, as identified in our previous article [24]. Particularly, we detail factors that need to be considered to enable collaborative practices which in turn are needed to implement BFHI [25]. While the relevance of inter-professional teamwork and collaboration has been emphasized by Schmied et al. [25] as well as other authors [17, 19, 20], our findings on professional rivalries and firmly established differences over childbirth go beyond these findings and mirror outcomes presented in the more general social science literature [31–33]. Similar to our findings, these studies reveal that effective collaboration is severely challenged by established approaches to childbirth and tensions over professional jurisdictions.

Furthermore, we showed that differing strategies health professionals pursued by their engagement in BFHI implementation impeded inter-professional teamwork and collaboration. Although nurses and midwives in this study shared the perception that their engagement in BFHI is an opportunity to advance their ‘professional project’ [34], nurses, in particular, considered the BFHI to be an opportunity to safeguard and protect their work vis-à-vis midwives and physicians. Midwives, however, considered BFHI complementary to their natural birth approach and, therefore, insisted that BFHI needs to be interpreted against the background of their naturalistic and empowering philosophical stance. As a consequence midwives and nurses are discordant on *how* to implement the BFHI. By contrast to nurses and midwives, physicians tried to defend their existing professional territory and passed along BFHI-related tasks to nurses and midwives. Physicians did not feel that BFHI brings about advantages for their professional project. These findings also mirror the results of a recently published article that explores the challenges of re-orientating hospitals towards health promotion. The authors argue that nurses as compared to physicians are more likely to implement health promotion programs because nurses consider engagement in health promotion as a strategy for expanding their professional jurisdictions [35].

Another factor that impeded collaboration and thus, the implementation of BFHI in a collaborative manner was the spatial constraints of maternity units. Especially midwives and nurses emphasized that the spatial division of maternity units hindered collaboration, particularly with regard to the timely exchange of information such as breastfeeding positions that were already shown in the labor room. Data also indicated that the spatial division let to a perceived separation between midwives and nurses. In accordance with previous BFHI studies [15–17, 25], the perceived separation became manifest in health professionals making an “inside-outside division”. Furthermore, interviewees argued that not only the spatial division impeded the timely sharing of knowledge among different professional groups, but data also revealed that interviewees struggled to collaborate due to the absence of continuous inter-professional team meetings. Thus, although the BFHI division in Austria recommends the establishment of an inter-professional project group to facilitate implementation [13], our findings indicate that further resources are required to establish inter-professional team meetings over time and thus, to facilitate collaboration.

In addition, our analysis revealed that collaboration was hindered because of midwives and physicians referring to their professional autonomy [34]. This enabled them to scrutinize and resist certain aspects of the BFHI. Midwives, for example, were concerned that naturalness would get lost due to introduction of skin-to-skin contact after c-section, whereas physicians perceived BFHI as an intrusion into their professional jurisdiction. Anesthesiologists, for example, argued that skin-to-skin practices as emphasized by BFHI hindered them in monitoring mothers’ skin. By contrast, nurses, due to a perceived lack of such autonomy, followed BFHI requirements quite strictly. Nurses’ strict adherence to BFHI requirements, versus midwives’ individual interpretation and physicians’ circumvention strategies resulted in several arguments, all aggravating the built-in difficulties of inter-professional teamwork and collaboration.

Our data indicates that it is the triad of midwives, nurses, and physicians that has to be considered when analyzing collaborative practices along with BFHI implementation on maternity units. The findings show that all professional groups are engaged in boundary work and draw on various discourses to legitimize their expertise and authority and demarcate their distinctive territories. In the more general literature, the phenomenon of distinctive professional territories is often discussed while referring to professionals’ ‘silo-thinking’. Frenk et al. [36] argue that genuine inter-professional collaboration is hardly part of the everyday work of health professionals. Our findings support the assumption that on maternity units as well, silo-thinking can be considered to be one fundamental barrier to effective inter-professional teamwork and collaboration and thereby, impedes the integration of BFHI into practice. This might be one explanation why the lack of inter-professional teamwork and collaboration was identified as considerable barrier to BFHI implementation in our previous work [24]. To break down silo-thinking among health professionals, Frenk et al. [36] emphasize that cooperation and collaboration competencies should become incorporated into the education of health professionals. Similarly, Sottas et al. [37] refer to the relevance of ‘transformative learning’ that emphasizes flexibility in thinking and acting in addition to teaching professional qualities and skills. The authors argue that, for example, a health campus would facilitate inter-professional teamwork and collaboration because, on such a campus, health professionals would be jointly educated from the beginning of their professional training. However, this rather represents a future direction and has not been established so far, at least not in Austria. Therefore, additional strategies to deal with these challenges on an organizational level are required to enable collaboration and inter-professional teamwork, and, ultimately, the implementation of BFHI. Based upon the findings of this study and our previous research [24], short-term facilitators for collaboration and inter-professional teamwork on maternity units can include:

- **Inter-professional exchange of monitoring and documentation data**: Monitoring and documenting the specific concerns of professionals that, for example, hinder skin-to-skin contact after c-section, as well as the attitudes and interpretations of particular BFHI-content among professional groups, can help to create a mutual understanding of the BFHI and facilitate collaboration [24]. This strategy can help to overcome the problem of timely sharing of knowledge. Yet, emphasis should be placed on sharing this information on an inter-professional basis. For example, in line with findings from Walsh et al. [17], documenting reasons for the provision of formula feeding should be discussed, at least among nurses and physicians, as they are currently those who are responsible for mother-baby dyads on the ward.
- **Regular inter-professional team meetings**: The BFHI in Austria recommends the establishment of an inter-professional BFHI project group. This can be used as a starting point to establish regular inter-professional team meetings whose lack was emphasized by our interviewees. Such meeting could help to share perceptions of and experiences with BFHI-related activities. Further research is needed, to investigate how such meetings can be established and sustained over time.
- **Inter-professional training activities**: Training of health professional represents a fundamental prerequisite for maternity units to achieve BFHI-certification. The relevance of training to facilitate BFHI implementation has been emphasized previously [24, 38]. The Austrian BFHI certification authority defined the scope of training as 20 h for midwives and nurses, 10 h for physicians and 4 h for nursing assistants working in the maternity units. However, our data indicated that professionals remain within their professional jurisdictions and experience difficulties to overcome these jurisdictions in order to collaboratively implement BFHI. One option to make professionals familiar with BFHI-related activities and collaborative practices are inter-professional training activities. Such training activities should be used as an opportunity to discuss professionals’ different approaches to childbirth and breastfeeding as well as ways to integrate BFHI. It is important to stress that training activities should pay particular attention to enabling midwives, nurses, and physicians to learn how to work together and how to collaboratively integrate the BFHI into practice. A BFHI-simulation training can probably enhance current training activities. In this respect, Watters et al. [39] have shown that inter-professional simulation training of midwives, nurses, and physicians in the UK enhances inter-professional communication and teamwork.

### Methodological considerations

The strength of this study is that it involved a wide range of health professionals with backgrounds in midwifery and nursing, as well as medicine from three different hospitals in Austria. The qualitative approach provided valuable insights into professionals’ conceptual understanding and perception of childbirth and breastfeeding as well as struggles towards collaborative practices on maternity units. However, the study was limited to three urban hospitals already involved in BFHI implementation in one province of Austria. Therefore, the findings have to be interpreted with caution and further studies are needed to investigate how our findings translate to other hospitals in Austria and beyond. We also have to assume that only professionals who are quite interested and competent in BFHI participated in this study because participation depended on professionals’ availability as well as their voluntariness. Accordingly, we might have missed more critical perceptions.

## Conclusions

The findings suggest that inter-professional teamwork and collaboration on maternity units is considerably limited by diverging approaches to childbirth and breastfeeding as well as deep-seated professional jurisdictions, spatial constraints, and ultimately, the different professions pursued diverging strategies when engaging in the BFHI. Effective working together and good collaborative practices among midwives, nurses, and physicians require extensive and continuous investments in their skills and competencies. While inter-professional teamwork and collaboration is a widely espoused goal of contemporary healthcare, this article has shown that it is of considerable importance to investigate how collaboration and related struggles between professions become evident within social practices in a specific field of healthcare. Especially in the face of implementing BFHI, creative solutions to bringing together different perspectives and taking advantage of the range of knowledge and practices from the different health professionals should be investigated.

## Additional file

Additional file 1: Interview guide. (DOCX 29 kb)

## Abbreviations

BFHI: Baby-Friendly Hospital Initiative
WHO: World Health Organization
UNICEF: United Nations Children’s Fund
