# Supplementary material for: The struggle for inter-professional teamwork and collaboration in maternity care: Austrian health professionals’ perspectives on the implementation of the Baby-Friendly Hospital Initiative
Source: BMC Health Serv Res. 2016 Mar 14;16:91. doi: 10.1186/s12913-016-1336-3 (PMC4791969; doi:10.1186/s12913-016-1336-3)
Supplement: Additional file 1: — Interview guide. (DOCX 29 kb) [file 12913_2016_1336_MOESM1_ESM.docx]

## Interview guide

**Introduction**

- Welcome and introduction of interviewers with names
- Thanking for participation in interview
- Clarification of time schedule (max. 1 hour)
- „Framing“ of study / content of interview
  - Our research interest is how the Baby-Friendly Hospital Initiative (BFHI) is implemented in maternity units. By implementation we mean how the BFHI-requirements become part of daily work routines in maternity units. To better understand this process, we collect different perspectives and experiences of health professionals (midwives, nurses, and physicians) that are working in different maternity units in one federal state of Austria. Finally, this study aims to explore facilitating and hindering factors with regard to BFHI implementation in Austria.
- Request to audiotape the interview; guarantee that all interviews will be anonymized and confidentiality will be ensured at all stages of the research process; clarification that audiotape can be switched off at any time
- Signature of written informed consent form
- **Start audiotape**

**Personal information**

**Q1: Please describe your position and range of tasks in this maternity unit.**

Possible follow-up questions:

- Specify how long have you been working in this maternity unit.
- Outline whether you have been working in other maternity units before.

1. **Selection of BFHI**

**Q2: Please describe why your maternity unit has decided to become a Baby-Friendly Hospital.**

Possible follow-up questions:

- Further specify reasons / motives for selecting BFHI (relevance to the organization, the staff, and mothers).
- Describe by whom the decision was taken, i.e. who was involved in the decision-making process.
- Describe how you and your colleagues have been involved.

1. **Installation of BFHI**

**Q3: Please describe the next steps once the decision was taken to become BFHI-certified.**

Possible follow-up questions:

- Please further elaborate on persons that were particularly involved in the installation of BFHI.
- Specify your own role in this process.
- Please describe the distribution of responsibilities.
- Please describe available support structures, e.g. additional financial or time resources.
- Please asses the effort to prepare bringing BFHI into daily work routines of your maternity unit (for you, for the maternity unit, for mothers).

1. **Operation of BFHI**

**Q4: Please describe how BFHI has changed your daily work routines.**

Possible follow-up questions:

- Specify / provide examples how BFHI requirements become enacted in practice / become implemented in daily work routines.
- Describe your own experience with implementing BFHI in daily work routines.
- Describe how your colleagues experience the implementation of BFHI.

1. **Consequences of BFHI operation**

**Q5: Please share your current conclusion with regard to the implementation of BFHI.**

1. For you as an employee.
2. For your daily routines and working together with colleagues.
3. For mothers and babies.
4. **Reflections on program implementation in hospitals**

**Q6: Please describe major factors that are crucial for successful implementation of BFHI.**

Possible follow-up questions:

- Please describe how implementation could be optimized / more easily facilitated.

**Q7: Please describe whether your organization has experience in implementing other, similarly complex programs?**

**Closing**

**Q7: Do you want to add anything?**
